# Does a ketogenic diet have beneficial effects on quality of life, physical activity or biomarkers in patients with breast cancer: a randomized controlled clinical trial

**DOI:** 10.1186/s12937-020-00596-y

**Published:** 2020-08-22

**Authors:** Adeleh Khodabakhshi, Thomas N. Seyfried, Miriam Kalamian, Maryam Beheshti, Sayed Hossein Davoodi

**Affiliations:** 1 Department of Nutrition, School of Public Health, Kerman University of Medical Sciences, Kerman, Iran; 2 Physiology Research Center, Kerman University of Medical Sciences, Kerman, Iran; 3 Biology Department, Boston College, Chestnut Hill, MA USA; 4 Dietary Therapies LLC, Hamilton, MT USA; 5 Department of Nutrition and Dietetics, Mofid children’s hospital, Shahid Beheshti University of Medical Sciences, Tehran, Iran; 6 Cancer Research Center, Shahid Beheshti University of Medical Sciences, Tehran, Iran; 7 Department of Cellular and Molecular Nutrition, Faculty of Nutrition Science and Food Technology, Shahid Beheshti University of Medical Sciences, Tehran, Iran

**Keywords:** Ketogenic diet, Breast cancer, quality of life, Physical activity, Lactate, Alkaline phosphatase, chemotherapy

## Abstract

**Introduction:**

Despite recent interest in the use of ketogenic diets (KDs) for cancer**,** evidence of beneficial effects is lacking. This study examined the impact of a randomly assigned KD on quality of life, physical activity and biomarkers in patients with breast cancer.

**Method:**

A total of 80 patients with locally advanced or metastatic breast cancer and without a history of renal disease or diabetes were randomly assigned to either a KD or a control group for this 12-week trial. Concurrent with the first, third, and fifth chemotherapy sessions, quality of life, physical activity, and biomarkers (thyroid function tests, electrolytes, albumin, ammonia, ALP, lactate and serum ketones) were assessed. Dietary intake was also recorded on admission and the end of the treatment.

**Results:**

No significant differences were seen in quality of life or physical activity scores between the two groups after 12 weeks; however, the KD group showed higher global quality of life and physical activity scores compared to the control group at 6 weeks (*P* = 0.02 *P* = 0.01). Also, serum lactate and ALP levels decreased significantly in the KD group compared to the control group at the end of the intervention (10.7 ± 3 vs 13.3 ± 4, 149 ± 71 vs 240 ± 164, *P* = 0.02 and *P* = 0.007, respectively). A significant inverse association was observed between total carbohydrate intake and serum beta-hydroxybutyrate at 12 weeks (r = − 0.77 *P* < 0.001). No significant differences between groups were observed in thyroid hormones, electrolytes, albumin, LDH or ammonia. Compliance among KD subjects ranged from 66.7 to 79.2% as assessed by dietary intake and serum ketones levels of > 0.5.

**Conclusion:**

According to our results, besides a higher global quality of life and physical activity scores compared to the control group at 6 weeks, KD diet combined to chemotherapy in patients with breast cancer does not bring additional benefit about quality of life and physical activity at 12 weeks. However, decreases seen in levels of lactate and ALP in the KD group suggest that a KD may benefit patients with breast cancer.

**Trial registration:**

This trial has been registered on Iranian Registry of Clinical Trials (IRCT) under the identification code: IRCT20171105037259N2 https://www.irct.ir/trial/30755

## Introduction

Ketogenic diets (KDs) are high in fat and very low in carbohydrate. They have been used as a dietary treatment in epilepsy for nearly a century [1]. Recently, KDs have gained the attention of cancer researchers due to their potential impact on cancer cell metabolism [2]. Despite the growing evidence of possible anti-tumor benefits, there are still some concerns about potential adverse effects of KDs in cancer patients, including micronutrient deficiencies, appetite reduction, nausea, constipation [3], fatigue [4], hyperlipidemia and especially unintended weight loss [3, 5]. KDs are perceived as restrictive in nature which may add to the burden of cancer patients who already suffer from considerable physical, emotional, and financial stress, all of which are known to negatively impact quality of life (QoL). In addition, alterations in physical and cognitive function during cancer treatment are pervasive. It is estimated that 25–99% of patients undergoing cancer treatment suffer from cancer-related fatigue [6]. Prior studies have found that KD may improve physical and mental well-being [7]. Less fatigue has been reported in healthy overweight and obese adults following low-glycemic compared to high-glycemic diets [8]. Results of three studies using the validated European Organization for Research and Treatment core QoL questionnaire to assess fatigue lacked consistent findings [9–11]. A small trial in advanced cancer patients showed improvement in sleep and emotional function after a three-month KD intervention [12]. Other studies have suggested enhanced cognitive function [9, 13].

To date, only four studies have assessed QoL in adult patients with cancer [7, 9–11]. Hunger is a reported side effect of restricted KDs; however, previous research has found that perceived hunger is reduced in low carbohydrate diets compared to low fat diets [14]. A recent systematic review has highlighted the need for additional, larger investigations on the impact of ketogenic diets on QoL [15]. The goal of this present trial was to assess whether a KD had beneficial effects on QoL, dietary intake, physical activity, and specific biomarkers in individuals with breast cancer while also evaluating compliance to KD guidelines in these patients.

The protocol used in this trial [16] and part of the results from this trial have been previously published [17, 18].

## Methods

The study protocol was approved by the National Nutrition and Food Technology Research Institute (NNFTRI), Shahid Beheshti University of Medical Sciences (SBMU), Tehran, Iran (IR.SBMU.NNFTRI.REC.1396.187). All participants provided written informed consent prior to participating in the study.

This trial was a randomized controlled open-label clinical trial open to breast cancer patients with locally advanced or metastatic disease who were receiving chemotherapy for at least 12 weeks. The study was conducted at the medical oncology clinic at Shohada-e-Tajrish hospital, Cancer Research Center, Tehran, Iran, from July 2017 to October of 2018. Participation was open to patients 18 to 70 years of age. Exclusion criteria screened for significant cardiac, renal or neurologic comorbidities; symptoms of malnutrition, diabetes, pregnancy, and Karnofsky index less than 70. Using a block balanced randomization method, patients were assigned to the intervention (*n* = 40) or control (n = 40) groups. Randomization was computer-generated by a statistician who was not a member of the medical team. Blinding the participants or study personnel was not deemed feasible in this diet intervention. The project coordinator enrolled the participants and assigned them to their interventions. Both the KD and the control diet were calculated to be eucaloric using the Mifflin-St. Jeor formula. The KD consisted of 6% of calories from CHO, 19% from protein, 20% from medium-chain triglyceride (MCT) oil, and 55% from fat. A dietitian provided specific nutritional counseling to each participant in individual face-to-face meetings. Patients engaged in ongoing weekly counseling sessions via phone, WhatsApp, or Telegram and were assessed for compliance and possible adverse effects. To further enhance compliance, dietary recommendations were individualized and appropriate recipes were provided to patients in the KD group were asked to refrain from eating any grains, grain products, starchy vegetables, fruit or sugar. Dietary carbohydrates were limited to non-starchy vegetables, and dietary proteins were obtained primarily from egg, meat, poultry and fish. Small amounts of lower carbohydrate berries and nuts were allowed as long as they did not exceed the carbohydrate limit in the diet prescription. Subjects were encouraged to increase their fat intake and to select from a variety of sources, including olive oil, butter and cream cheese. Patients were asked to choose only the foods specified in the diet plan provided to them. Patients were also encouraged to use medium-chain triglyceride (MCT) oil. MCT oil, an odorless and tasteless saturated fat, does not require bile or pancreatic enzymes for digestion. It is easily converted to ketones in the liver thereby enhancing ketosis. Every 2 weeks, 500 ml of MCT oil from Nutricia (Erlangen, Germany) was provided to each subject in the KD group. For better tolerance, initial dosage of MCT was kept low and increased daily over a 6-day period until maximum tolerable dosage was achieved. Dosage was reduced in a similar stepped process.

The patients in the control group were instructed to follow a standard diet consisting of 55% CHO, 15% protein, and 30% fat. Dietary compliance was checked by assessing blood beta-hydroxybutyrate levels every 3 weeks and dietary intake at baseline and end of the study.

### QoL assessment

QoL was assessed using the EORTC QLQ-C30 (version 2) and IORTC QLQ-BR23 questionnaires developed by the European Organization for Research and Treatment of Cancer. The validity and reliability of the questionnaires has previously been evaluated in Iran [19, 20]. The questionnaires were completed at enrollment, at 6-weeks, and at the end of the intervention.

### Dietary intake assessment

Hospital dietitians used a 24-h dietary recall (24HR) to obtain a total of 3 days intake (one weekend day and two workdays) through telephone and face-to-face interviews both at the beginning and end of the study. The amount of each food consumed was estimated using common household containers (bowls, cups, and glasses) and standard measuring cups and spoons as references. The mean quantity of total energy, carbohydrate, protein and fat were estimated from the 24HRDietary intake was analyzed by Nutritionist IV software (Version 3.5.2 US).

#### Physical activity assessment

Physical activity was measured using the IPAC (International Physical Activity) questionnaire at baseline, at 6 weeks, and at the end of the study.

#### Biomarker assessment

Fasting blood sampling for serum Na^+^, K^+^, Ca^++^, P^+^, lactate, Mg^++^, LDH, albumin, ammonia, and ALP were performed at baseline, midway through the intervention (6 weeks), and at 12 weeks. T_3_, T_4_, and TSH were measured at baseline and the end of the intervention.

### Statistical analysis

Considering the 80% power and α = 0.05, the sample size was calculated as 30 individuals per group. Assuming a 20% dropout during the 12 weeks of the study, the final number of participants was calculated as 40 patients in each group.

Statistical analysis was carried out according to the intention-to-treat protocol. Continuous variables were tested for normal distribution by the Kolmogorov-Smirnov test and then reported as mean ± standard deviation or median as appropriate. Student t-test or Mann–Whitney U test was used to compare the continuous variables between the two groups. Paired sample t-test or Wilcoxon was used to compare the continuous variables within the two groups. The ANCOVA test was used to eliminate the effect of confounding factors.

Pearson correlation analyses were used to estimate associations between total carbohydrate intake and serum beta-hydroxybutyrate.

Data were analyzed using the SPSS version 18.0 software (Chicago, IL, USA) and Stata version 13. *P* < 0.05 was considered as statistically significant.

## Results

Detailed patient demographics and a flow diagram were reported previously [17]. A total of 80 women with breast cancer were enrolled and randomly assigned to either the intervention (*n* = 40) or control (n = 40) groups. Three patients in the control group withdrew before beginning their assigned diet, while10 patients in the KD group and 10 patients in the control group withdrew from the study after beginning their assigned diet. Ultimately, 30 patients in each group completed the study and were included in the analysis. No significant differences were seen between the two groups with regard to age, cancer type, metastasis, and marriage or education status (*P* > 0.05). The intervention group included 25 patients with locally advanced disease and 5 patients with metastatic disease (1 liver, 1 bone, 1 lung, 2 liver and bone) while the control group consisted of 19 patients with locally advanced disease and 11 patients with metastatic disease (6 bone, 1 liver, 1 lung, 1 liver and bone, 2 at other sites) (*P* = 0.08) Table 1.

**Table 1:** Baseline characteristics in breast cancer patients before intervention

	Scale categories	Intervention (Ketogenic diet) ***n*** = 30	Control (Ordinary) ***n*** = 30	***p***- value
Cancer Type	Locally Advanced	25 (83.3)	19 (63.3)	0.08^a^
	Metastatic	5 (16.7)	11 (36.7)	
ER	positive	22 (73.3)	20 (66.7)	0.57^a^
	negative	8 (26.7)	10 (33.3)	
PR	positive	15 (50)	18 (60)	0.43^a^
	negative	15 (50)	12 (40)	
HER2	positive	12 (40)	13 (43.3)	0.79^a^
	negative	18 (60)	17 (56.7)	

*ER* Estrogen receptor, *PR* Progesterone receptor

*HER2* Human epidermal growth factor receptor 2

^a^Calculated by chi square test

^b^Categorical data shown as No (%)

Data related to quality of life are shown in Tables 2, 3 and 4.

**Table 2:** Quality of life in breast cancer patients’ before and after intervention in KD group and control group as measured by the EORTC QLQ-C30^a^

Functioning^a^	KD	Control	MD (95% CI)	***p***-value
Physical functioning				
Week 0	89 ± 11^a^	76 ± 20	13 (4.4,21.7)	0/004
Week 12	78 ± 19	68 ± 20	9.9 (−.7,20)	0/06
*p*-value	0/04	0/05		
Role functioning				
Week 0	86 ± 16	79 ± 28	7.2 (−4.9,19.4)	0/24
Week 12	75 ± 25	66 ± 29	8.9 (−6,23)	0/23
*p*-value	0/10	0/02		
Cognitive functioning				
Week 0	85 ± 16	71 ± 28	13.7 (1,26)	0/03
Week 12	75 ± 19	72 ± 21	5.5(−8,14)	0/59
*p*-value	0/03	1		
Emotional functioning				
Week 0	67 ± 21	66 ± 21	1.1 (−9.9,12.2)	0/84
Week 12	62 ± 23	60 ± 21	2(−10,14)	0/73
*p*-value	0/34	0/11		
Social functioning				
Week 0	94 ± 17	93 ± 17	0.36(−8.8,9.5)	0/93
Week 12	91 ± 17	87 ± 17	3.5(−4.6,5.9)	0/45
*p*-value	0/38	0/02		
Global quality of life				
Week 0	68 ± 16	65 ± 16	3.6 (−5.1,12.3)	0/41
Week 12	70 ± 20	62 ± 20	8.1(−5.7,3.3)	0/16
*p*-value	0/64	0/49		

After adjusting for baseline value and chemotherapy status no significant differences were observed

Student t-test was used to compare the continuous variables between the two groups. Paired sample t-test was used to compare the continuous variables within the two groups

Data shown as mean and SD

^a^The higher values indicate higher level of functioning and quality of life

**Table 3:** Quality of life in breast cancer patients before and after intervention in KD group and control group as measured by the EORTC QLQ-C30

Symptoms^a^	KD	Control	***p***-value
Fatigue			
Week 0	22(8–33)^a^	33(11–44)	0/10
Week 12	33(19–55)	33(33–55)	0/66
*p*-value	0/01	0/02	
Nausea and vomiting			
Week 0	0(0–0)	0 (0–20)	0/21
Week 12	0(0–4)	16(0–16)	0/64
*p*-value	0/01	0/02	
Pain			
Week 0	16(0–50)	16(0–33)	0/59
Week 12	16(0–50)	33(16–50)	0/59
*p*-value	0/62	0/38	
Reduction in appetite			
Week 0	0(0–8)	0(0–33)	0.37
Week 12	33(0–33)	16(0–33)	0.48
*p*-value	0.02	0.41	
Sleep difficulties			
Week 0	16(0–33)	0(0–66)	1
Week 12	0(0–41)	33(0–50)	0/81
*p*-value	0/63	0/88	
Dyspnea			
Week 0	0(0–33)	0(0–33)	0/31
Week 12	33(0–41)	16(0–33)	0/43
*p*-value	0.05	0.73	
Constipation			
Week 0	0 (0–0)	0 (0–33)	0.24
Week 12	0 (0–33)	0 (0–33)	0.51
*p*-value	0/18	0/39	
Diarrhea			
Week 0	0 (0–0)	0 (0–0)	0.92
Week 12	0 (0–33)	0 (0–0)	0/65
*p*-value	0.71	0.20	
Financial concerns			
Week 0	0 (0–0)	0 (0–0)	0.49
Week 12	0 (0–33)	33 (0–58)	0.28
*p*-value	0.71	0.01	

Mann–Whitney U test was used to compare the continuous variables between the two groups. Wilcoxon was used to compare the continuous variables within the two groups

^a^The higher values indicate a higher grade of symptoms Data shown as median and quartile (25, 75)

**Table 4:** Quality of life in breast cancer patients before and after intervention in KD group and control group as measured by the EORTC QLQBR23^a^

	KD	Control	***p***-value
^a^Functioning			
Future perspective			
Week 0	66 (33–100)	66 (33–66)	0/60
Week 12	66 (33–66)	33 (33–100)	0/85
*p*-value	0/45	0/76	
^b^Symptoms+			
Arm			
Week 0	11 (0–36)	11 (0–22)	0/36
Week 12	11 (0–36)	22 (0–33)	88
*p*-value	0/70	0/59	
Breast			
Week 0	8 (0–33)	8 (0–25)	0/34
Week 12	8 (0–10)	8 (0–16)	0/55
*p*-value	0/01	0/34	
Systemic therapy side effects			
Week 0	9 (4–17)	14 (4–23)	0/54
Week 12	42 (20–52)	42 (33–52)	0/33
*p*-value	0</001	0</001	
Concerns over hair loss			
Week 0	0	0	0/20
Week 12	66 (33–100)	33 (33–100)	0/50
*p*-value	0</001	0</001	

Mann–Whitney U test was used to compare the continuous variables between the two groups. Wilcoxon was used to compare the continuous variables within the two groups

^a^The higher values indicate higher level of functioning and quality of life

^b^The higher values indicate a higher grade of symptoms

Data shown as median and quartile (25, 75)

No significant differences were seen in QoL between the two groups after 12 weeks; however, the KD group showed better global QoL compared to the control group at week 6 (*P* = 0.02).

Also at week 6 diarrhea increased in the control group compared to the intervention (*P* = 0.02). Data on week 6 not shown. Using the QoL questionnaire, there was a within-group decrease in reported hunger from baseline to 12 weeks in the KD group (*P* = 0.02). A within-group decrease was seen in physical performance measures from baseline to 12 weeks in both groups which was significant only in the KD group (*P* = 0.04). In addition, role functioning and social functioning scores significantly decreased in the control group compared to the baseline but not in the KD group (*P* = 0.02 P = 0.02) Table 2.

**Fig. 1:**
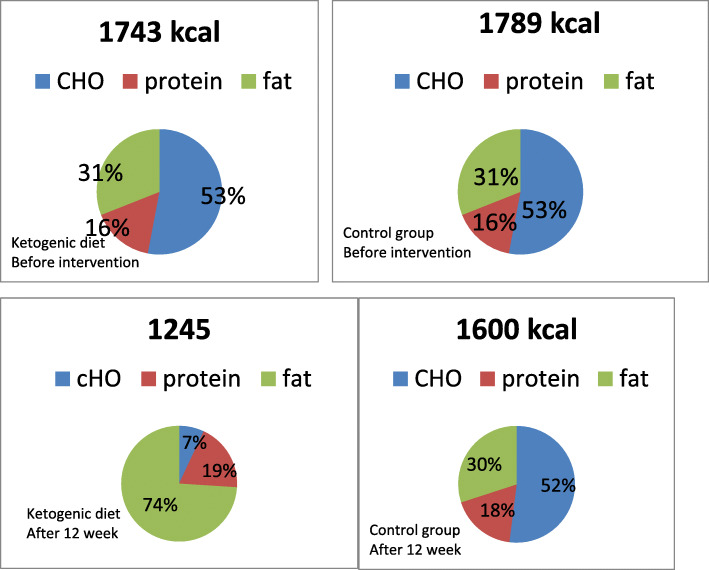
Mean caloric intake and distribution of macronutrients (as percentage of total kilocalories) before and after 12 week intervention, in breast cancer patients in two groups

Mean dietary intake is shown in Table 5 and Fig. 1. The mean caloric and carbohydrate intake decreased significantly at the end of the study compared to control (*P* = 0.003 and *P* < 0.001, respectively), while fat intake increased significantly in the KD group compared to the control group (*P* < 0.001). After adjusting for total energy intake, this difference remained significant. When data from both groups was combined, a significant inverse association was observed between total carbohydrate intake and serum beta-hydroxybutyrate at 12 weeks (*r* = − 0.77 *P* < 0.001), although this effect was not seen when the KD group was analyzed separately.

**Table 5:** Comparison of mean ± SD macronutrient intake at baseline and 12-weeks

Variable	KD Mean ± SD	Control Mean ± SD	MD (0.95 CI)	*p*-value
Energy (Kcal/day)				
Before	1743 ± 305	1789 ± 323	−45 (− 222,131)	0/60
After	1245 ± 360	1600 ± 304	− 355 (− 577,-132)	0/003
*p-*value	0</001	0/001		
Carbohydrate (gr)				
Before	235 ± 52	238 ± 54	−2.6 (−32,27)	0/85
After	22 ± 11	208 ± 60	− 185 (− 210,-159	0</001^a^
*p-*value	0</001	0/03		
Protein (gr)				
Before	73 ± 13	71 ± 22	1.5 (−8.9,11.9)	0/76
After	61 ± 61	72 ± 71	−10 (−21.9,1.5)	0.41^a^
*p*-value	0/02	0/003		
Fat (gr)				
Before	56 ± 11	61 ± 15	−4.6(−12.2,3)	0/23
After	101 ± 32	53 ± 11	48 (31,65)	0</001^a^
*p*-value	0</001	0/007		

Student t-test was used to compare the continuous variables between the two groups. Paired sample t-test was used to compare the continuous variables within the groups

*MD* Mean difference

*CI* Confidence interval

^a^Ancova: Adjusted for baseline value and energy

Within-group analysis showed significant decreases in energy, carbohydrate, and protein intake in both groups compared to the baseline, Fat intake increased significantly compared to the baseline in the KD group (*P* < 0.001) and decreased significantly in the control group (*P* = 0.007).

During the intervention, 96% of the subjects in the KD arm limited carbohydrates to < 50 g and 79.2% of subjects consumed < 10% of calories from carbohydrates.

At 12 weeks, 66.7% of patients in the KD group had serum ketones > 0.5 mmol/L; at 6-weeks, 70.4% had ketone levels of > 0.5 mmol/L. As previously reported serum ketone concentrations increased significantly in the KD group (0.007 ± 0.026 to 0.923 ± 0.699 mmol/l, *P* < 0.001) [17].

**Fig. 2:**
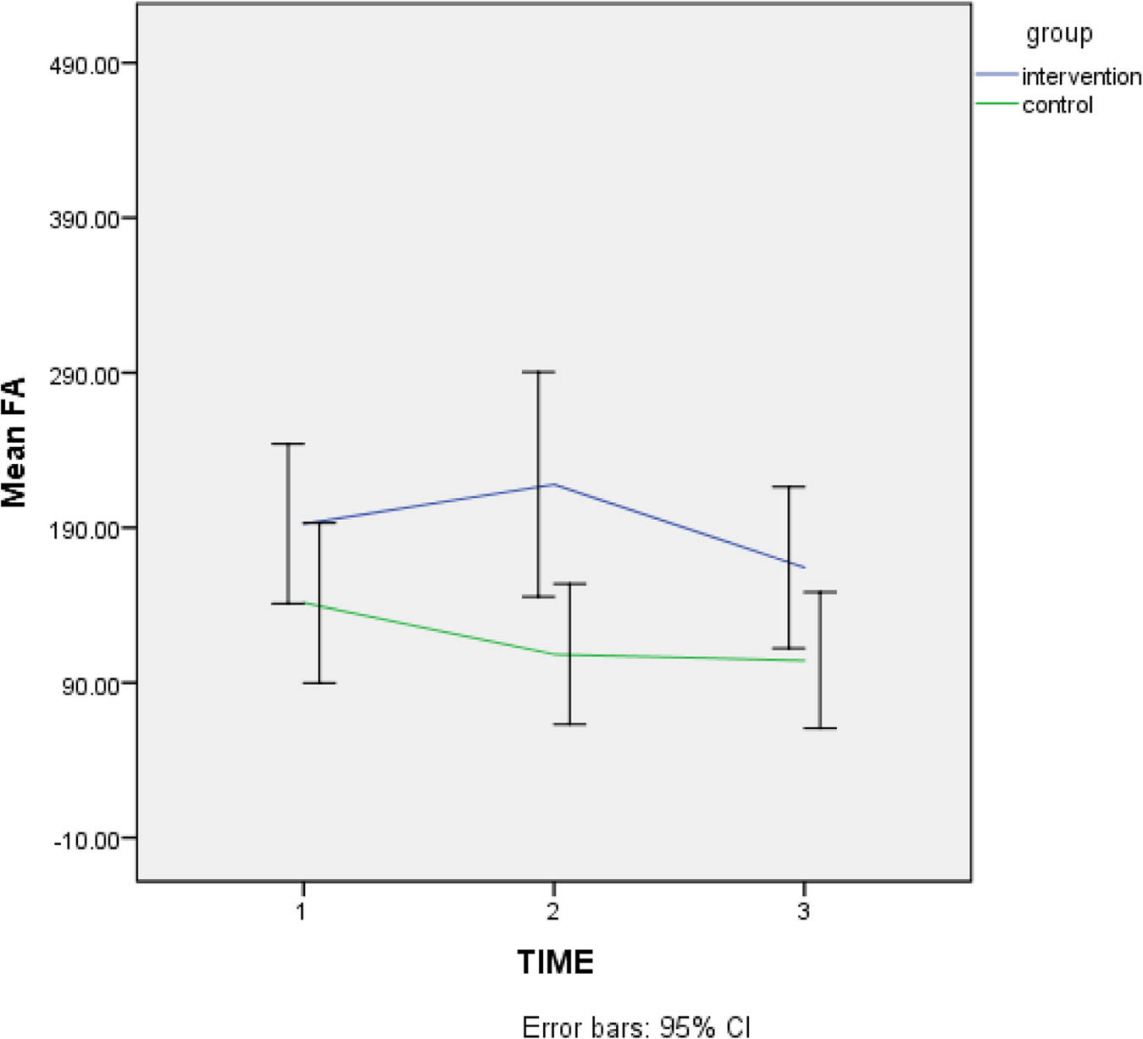
Comparison of trend changes in physical activity in breast cancer patients in two groups

At 6 weeks, physical activity improved in the KD group compared to the control group (adjusted for cancer type and baseline value *P* = 0.01) but after 12 weeks, physical activity did not show any significant differences in a between- or within-group analysis. (Fig. 2).

No significant difference was observed in a between- or within-group analysis of thyroid hormones, electrolytes, albumin, Ammonia, and LDH. However, lactate and ALP decreased significantly after intervention in the KD group compared to the control group (*P* = 0.02 and P = 0.02, respectively). ALP is adjusted for baseline value and cancer type. Table 6. Data on thyroid hormones not shown.

**Table 6:** Biomarker levels in control and KD-treated breast cancer patients

Variable	Trial Arms	Baseline	Midpoint	12 weeks	*p*- value midpoint vs BL	*p*-value 12wks vs midpoint	*p*-value 12wks vs BL
Lactate	KD	13.3 ± 11	10.8 ± 4	10.7 ± 3	1	1	1
	Control	13.8 ± 4	14.7 ± 6	13.3 ± 4	0.94	0.93	1
	MD (95% CI)	−0.51(−5.3,4.2)	−3.8 (−7,-0.7)	−2.6 (−4.8,-0.35)	1	0.01	0.02
LDH (u/l)	KD	680 ± 643	666 ± 770	535 ± 517	1	0.48	0.58
	Control	706 ± 560	627 ± 556	472 ± 148	1	0.36	0.12
	MD (95% CI)	−25 (− 374,323)	39 (− 330,409)	62 (− 145,271)	1	1	1
Ammonia (mcg/dl)	KD	58 ± 17	60 ± 17	66 ± 15	1	1	0.69
	Control	75 ± 27	71 ± 36	75 ± 24	1	0.92	1
	MD (95% CI)	−17 (−30,3.9)	−11(−29,6.9)	−9 (−20.8,2.4)	0.03	0.43	0.49
Albumin (g/dl)	KD	4.5 ± 0.33	4.5 ± 0.26	4.5 ± 0.33	1	1	1
	Control	4.4 ± 0.43	4.5 ± 0.37	4.5 ± 0.37	1	1	1
	MD (95% CI)	0.08(−0.13,0.29	0.04-(0.15,0.24)	0.04 (−0.16,0.2)	1	1	1
ALP	KD	197 ± 126	169 ± 76	149 ± 71	1	1	0.99
	Control	325 ± 375	333 ± 440	240 ± 164	1	0.11	0.51
	MD (95% CI)	− 127(− 285,29)	−164(−372,44)	−90(− 162,-19)	0.19	0. 13*	0.02*
K (meq/l)	KD	4.2 ± 0.39	4.1 ± 0.43	4.1 ± 0.33	1	0.46	0.13
	Control	4.2 ± 0.35	4.3 ± 0.38	4.2 ± 0.37	1	1	0.75
	MD95% CI	−0.05 (− 0.26,0.15)	−0.14)0.41,0.12(	−0.10 (−0.29,0.08)	1	0.69	0.88
P (mg/Dl)	KD	4 ± 0.71	3.9 ± 0.66	4.4 ± 0.88	1	0.05	0.06
	Control	4.1 ± 0.92	4.2 ± 0.52	4.3 ± 0.65	1	1	1
	MD95% CI	−0.17 (− 0.63,0.28(	−0.24 (0.63,0.14)	0.07 (−0.34,0.50(	1	0.93	1
Mg (mg/dL)	KD	2.1 ± 0.19	2 ± 0.13	2 ± 0.19	1	1	1
	Control	2.1 ± 0.21	2 ± 0.19	2 ± 0.23	0.43	0.82	1
	MD95% CI	−0.001 (− 0.11,0.11)	0.07 (− 0.03,0.18)	−0.02 (−0.14,0.09)	1	0.76	1
Ca (mg/dl)	KD	9.4 ± 1.2	9.4 ± 0.80	9.3 ± 0.59	0.37	1	0.16
	Control	9/4 ± 0.95	9.2 ± 0.78	9.4 ± 0.56	0.84	0.85	1
	MD95% CI	0.36	0.21	−0/10	0.42	1	1
Na (meq/l)	KD	146 ± 43	140 ± 3	140 ± 2	0.83	1	0.79
	Control	138 ± 2.8	138 ± 2	133 ± 24	1	0.80	0.80
	MD 95% CI	7.9(−9.8,25.7)	1.9 (0.19,3.6)	7 (−2.3,17.3)	0.56	1	0.63

*BL* Baseline, Midpoint: 1st follow-up or week 6, 12 weeks: 12 weeks or last follow-up, *MD* Mean Difference, *CI* Confidence Interval Analysis type: Repeated measure, all *p* values were calculated based on Bonferroni correction for multiple comparisons * Ancova: Adjusted for base line value and cancer type

## Discussion

The effect of KD on QoL, physical activity, dietary intake, and biomarkers in patients with locally advanced and metastatic breast cancers was evaluated in this study. Based on our findings, in the KD group, global QoL was higher at 6 weeks; perhaps in part because diarrhea was more frequent in the control group than the KD group. No significant differences were seen in the QoL, physical activity, and biomarkers between the two groups after the 12 week intervention. Lactate and ALP were lower in the KD group compared to the control.

### Effect of diet on QoL

In our study, in the KD group, global QoL was higher at 6 weeks. No adverse effects were observed in those participants assigned to the KD compared to the control group after 12 weeks. Within-group analysis showed decreased hunger and physical function in the KD group compared to the baseline. In the control group, role and social functioning decreased significantly compared to baseline.

Results of a systematic review and meta-analysis have shown that KDs suppress appetite [14]. Decrease in hunger or appetite in our study may be due to the high fat content of the KD as it decreases the ghrelin release which in turn may reduce appetite. High fat intake also slows digestion which could also impact the perception of hunger. Previously we have shown that the KD results in weight loss [17]. As a clinical benefit, KD-induced decreases in appetite, weight, and body fat may result in favorable changes in breast cancer patients, notably in overweight or obese women [21, 22].

In contrast with our findings, Cohen found that a KD significantly enhanced physical function scores in women with ovarian or endometrial cancer compared to the control group but appetite did not change at the end of the study compared to the baseline [7]. Part of the inconsistency between our study and Cohen’s trial may be explained by the design of the study. While only 25% of the participants in the Cohen study were undergoing chemotherapy, all of our patients were receiving treatment.

Also, timing of the administration of the questionnaires and whether the participants were in positive or negative energy balance may have influenced our findings.

No significant difference was reported in QoL at the end of study compared to the baseline by Tan-Shalaby et al. [23]. However, a slight decrease in physical and role functioning as well as temporary constipation and fatigue were reported in the KD group in one study [9]. In our study, constipation was noted by participants in the KD arm during the early days which was managed by dietary changes.

Also, after 6 weeks, in the KD group, physical activity scores was higher compared to the control group but at 12 weeks differences in scores were not significant between the two groups.

### Dietary intake and adherence

Our study data showed a significant decrease in carbohydrate intake and a significant increase in fat intake in the KD group compared to the control. Protein intake was not significantly different between the two groups but decreased overall in both groups when compared to baseline. Total daily carbohydrate intake was similar to results in the Cohen study [24]. We also assessed serum beta-hydroxybutyrate: In the KD group, 66.7% of patients at 12 weeks and 70.4% at 6-weekshad serum ketones > 0.5 and 89% patients at 6 weeks and 12 weeks had serum ketones > 0.3 mmol/l. Cohen reported that 57% of patients had beta-hydroxybutyrate concentrations > 0.5 mmol/l.

A recent systematic study of KDs in adult cancer patients reported a range of 23 to 100%, with a 49% adherence rate overall reported by [15]. According to our data, the level of adherence to the KD intervention suggests that the diet is a feasible option for women with breast cancer who are receiving chemotherapy.

Despite the lack of any restriction in calorie intake in the study design and consistent with findings of Cohen [25], the KD group showed a significant reduction in calorie intake compared to the control group. The decrease in calorie intake may be due to reductions in appetite associated with ketosis as the subjects in the KD arm did not consume all of the fat calculated for their diet. This may also be due in part to customary practices surrounding meal preparation. A decrease in appetite and subsequent inadvertent calorie restriction most often results in weight loss; in the absence of malnutrition or cachexia, this may have anti-inflammatory and pro-apoptotic properties which in turn may exert a positive effect on these hallmarks of cancer. Ketosis may also enhance the effectiveness of chemotherapy while reducing the side effects of treatment [26, 27].

### Effect of diet on biomarkers

Consistent with the outcomes of the previous studies, our results revealed that the KD had no adverse effect on thyroid hormones, electrolytes, LDH, urea, and albumin. Significant decreases were seen in serum levels of lactate. KDs reduce glycolytic activity which in turn may slow metastases by reducing the acidity of the tumor microenvironment and lowering the availability of lactate as a substrate for biomass synthesis [28]. Decreases were also seen in ALP: High levels of ALP in breast cancer patients is a negative prognostic marker, often indicating progression of metastatic disease [29]. More research is needed to assess whether lower ALP and lactate as seen in this study contributes to slower rates of disease progression.

To our knowledge, this is the first randomized controlled trial examining the effects of a KD on QoL in breast cancer patients.

The primary limitation of this study was the heterogeneous nature of the sample in regards to cancer stage. A secondary limitation was the small sample size.

## Conclusion

According to our results, besides a higher global QoL and physical activity scores compared to the control group at 6 weeks, KD diet combined to chemotherapy in patients with breast cancer does not bring additional benefit about QoL and physical activity at 12 weeks. While many blood biomarkers did not differ significantly between the two groups, ketosis may still offer benefit to some patients with breast cancer in part by decreasing lactate and ALP.

## Supplementary information

**Additional file 1: figure 1.** Flow diagram of the patient treatment process.

**Additional file 2: figure 2.** Median (confidence interval) tyroid hormones in baseline and 12-week by two trial arms in breast cancer patients.

## Data Availability

Data described in the manuscript, code book, and analytic code will be made available upon request pending.
