# Supplementary material for: Does a ketogenic diet have beneficial effects on quality of life, physical activity or biomarkers in patients with breast cancer: a randomized controlled clinical trial
Source: Nutr J. 2020 Aug 22;19:87. doi: 10.1186/s12937-020-00596-y (PMC7443288; doi:10.1186/s12937-020-00596-y)
Supplement: Supplementary file 1 — Additional file 1: figure 1. Flow diagram of the patient treatment process. [file 12937_2020_596_MOESM1_ESM.docx]

Allocated to KD (n= 40 )

## Follow-Up

Discontinued: 7

5: frequent blood sampling

1: surgery

1: diabetes after entering the study

Allocated to control (n= 40 )

Analysed (n= 30 ))

## Analysis

Analysed (n=30 )

## Enrollment

## Allocation

Randomized (n=80 )

Excluded (n= 27 )

♦  Not meeting inclusion criteria (n=14 )

♦  Declined to participate (n=10 )

♦  Other reasons (n= 3 )

Assessed for eligibility (n= 107 )

Did not enroll due to scheduling conflicts (3)

Did not accept assignmentl to the ketogenic diet (2)

Discontinued: 8

2:nausea and hypoglycemia( 50mg/dl)

3: weakness and hunger

2. unable to remain compliant to diet

2. lack of energy and oiliness of the diet

1. lack of energy and oiliness

**Additional figure 1: Flow diagram of the patient treatment process**

KD: Ketogenic diet
