# Supplementary material for: Does a ketogenic diet have beneficial effects on quality of life, physical activity or biomarkers in patients with breast cancer: a randomized controlled clinical trial
Source: Nutr J. 2020 Aug 22;19:87. doi: 10.1186/s12937-020-00596-y (PMC7443288; doi:10.1186/s12937-020-00596-y)
Supplement: Supplementary file 2 — Additional file 2: figure 2. Median (confidence interval) tyroid hormones in baseline and 12-week by two trial arms in breast cancer patients. [file 12937_2020_596_MOESM2_ESM.docx]

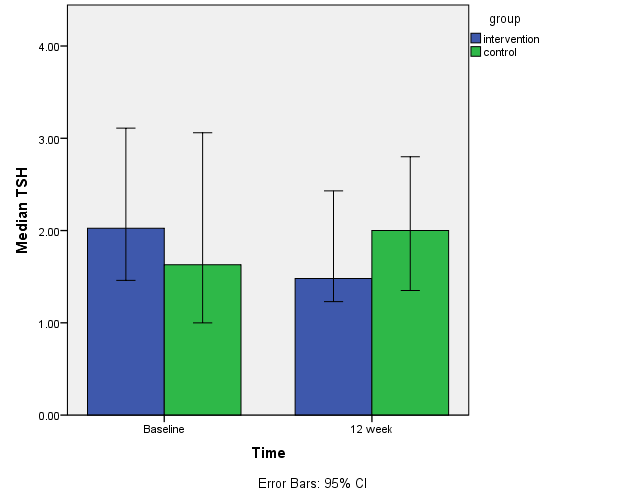

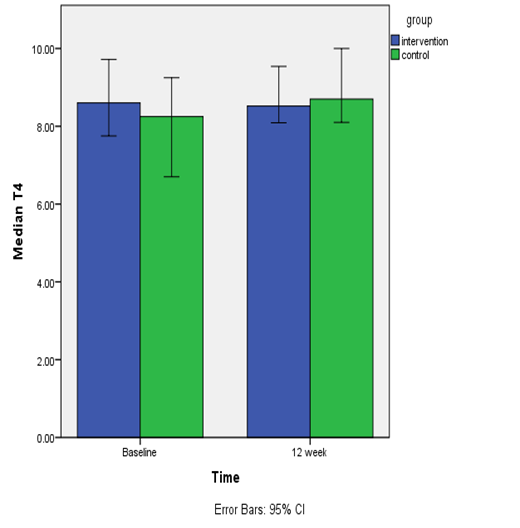


Error Bars: 95% CI


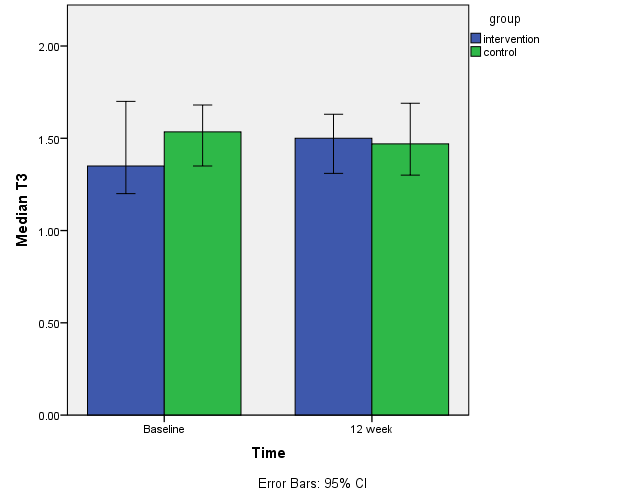


**Additional figure 2: Median (confidence interval) tyroid hormones in baseline and 12-week**

**by two trial arms in breast cancer patients**
